# CVW-Etr: A High-Precision Method for Estimating the Severity Level of Cotton Verticillium Wilt Disease

**DOI:** 10.3390/plants13212960

**Published:** 2024-10-23

**Authors:** Pan Pan, Qiong Yao, Jiawei Shen, Lin Hu, Sijian Zhao, Longyu Huang, Guoping Yu, Guomin Zhou, Jianhua Zhang

**Affiliations:** 1 Agricultural Information Institute, Chinese Academy of Agricultural Sciences, Beijing 100081, China; cn.kerrypan@gmail.com (P.P.); yaoqiong24@163.com (Q.Y.); alphajiawei@foxmail.com (J.S.); zhaosijian@caas.cn (S.Z.); zhouguomin@caas.cn (G.Z.); 2 National Agriculture Science Data Center, Beijing 100081, China; 3 National Nanfan Research Institute (Sanya), Chinese Academy of Agricultural Sciences, Sanya 572024, China; huanglongyu1@163.com (L.H.); yuguoping@caas.cn (G.Y.); 4 Agricultural College, Henan University, Kaifeng 475004, China; 5 Institute of Cotton Research, Chinese Academy of Agricultural Sciences, Anyang 455000, China; 6 China National Rice Research Institute, Hangzhou 311401, China; 7 Nanjing Institute of Agricultural Mechanization, Ministry of Agriculture and Rural Affairs, Nanjing 210014, China

**Keywords:** cotton verticillium wilt, crop disease severity level estimation, deep learning, MobileSAM, YOLOv8-Seg

## Abstract

Cotton verticillium wilt significantly impacts both cotton quality and yield. Selecting disease-resistant varieties and using their resistance genes in breeding is an effective and economical control measure. Accurate severity estimation of this disease is crucial for breeding resistant cotton varieties. However, current methods fall short, slowing the breeding process. To address these challenges, this paper introduces CVW-Etr, a high-precision method for estimating the severity of cotton verticillium wilt. CVW-Etr classifies severity into six levels (L0 to L5) based on the proportion of segmented diseased leaves to lesions. Upon integrating YOLOv8-Seg with MobileSAM, CVW-Etr demonstrates excellent performance and efficiency with limited samples in complex field conditions. It incorporates the RFCBAMConv, C2f-RFCBAMConv, AWDownSample-Lite, and GSegment modules to handle blurry transitions between healthy and diseased regions and variations in angle and distance during image collection, and to optimize the model’s parameter size and computational complexity. Our experimental results show that CVW-Etr effectively segments diseased leaves and lesions, achieving a mean average precision (mAP) of 92.90% and an average severity estimation accuracy of 92.92% with only 2.6M parameters and 10.1G FLOPS. Through experiments, CVW-Etr proves robust in estimating cotton verticillium wilt severity, offering valuable insights for disease-resistant cotton breeding applications.

## 1. Introduction

Cotton, an essential textile fiber from the Gossypium genus in the Malvaceae family, contributes to approximately 35% of global annual fiber demand [1]. Diseases affecting cotton throughout its growth cycle can significantly reduce yield and quality, posing a serious economic threat to farmers [2]. One of the most significant challenges in cotton production is verticillium wilt, a persistent disease since it was first documented in 1918 [3]. This highly destructive disease presents substantial obstacles to the growth and development of cotton due to its widespread distribution and formidable pathogenicity under favorable conditions [4]. Economic losses can reach alarming rates of 30–50% or more in certain years, largely attributed to inadequate prevention measures and misguided interventions [5].

In cotton cultivation, controlling verticillium wilt often relies on fungicides and fumigation [6,7]. While widely embraced by farmers and generally effective, it is costly, hazardous, and not environmentally friendly [8]. In contrast to fungicides and fumigation, selecting cotton varieties with strong disease resistance and utilizing their resistance genes in breeding can more effectively reduce losses caused by cotton verticillium wilt [9]. To develop cotton varieties with high verticillium wilt resistance, breeders establish experimental plots to estimate disease resistance in various genotypes and progeny lines [10]. During estimation, parameters like disease incidence, severity level, and the time it takes from planting or inoculation to symptom manifestation are considered [11]. Among these, accurately estimating disease severity level is pivotal at every stage, spanning from germplasm selection and progeny screening to variety dissemination.

Ensuring accurate and precise estimates of disease severity levels is crucial for developing cotton varieties with high disease resistance. However, the current manual method, which relies on visually scoring lesions on cotton leaves, is prone to estimator fatigue, bias, and errors, and is time-consuming [12]. Moreover, it requires experienced estimators, and different estimators may yield significantly varied estimations of the same sampling unit [13]. These challenges hinder the facilitation and acceleration of disease-resistance breeding processes. Given the growing need for the accurate and large-scale estimation of disease severity levels for cotton verticillium wilt-resistance breeding, the urgency for automated methods is escalating [14]. Significant attention has been devoted to achieving high-precision estimates of cotton verticillium wilt disease severity level [15].

In recent years, researchers have employed methods such as spectral analysis [16] and unmanned aerial vehicle (UAV) remote sensing to estimate cotton verticillium wilt disease severity [17,18]. However, their effectiveness in disease-resistance breeding is often limited due to the high environmental requirements of spectral measurement devices and the inability of UAV remote sensing to accurately estimate individual disease-resistant breeding materials. Hence, further research is crucial to develop more suitable methods tailored to the demands of disease-resistance breeding applications. The segmentation of diseased leaves and lesions, utilized to calculate the diseased leaf area and lesion area, is the predominant approach for estimating cotton verticillium wilt disease severity levels in disease-resistance breeding [19]. However, accurately segmenting diseased leaves and lesions under field conditions persists as a primary challenge for this estimation method [20].

Disease segmentation involves segmenting crop disease or lesion targets from complex backgrounds, comprising tasks such as leaf and lesion segmentation. Traditional image processing techniques [21,22], region growth algorithms [23], and machine learning [24,25] have been proposed for segmenting crop diseases over the past two decades. However, these methods are only effective for diseased-leaf images with simple backgrounds. When the color of the diseased area closely resembles the background or the boundaries are unclear, these segmentation methods struggle to differentiate between the background and the lesions on diseased leaves, resulting in poor segmentation outcomes.

Deep learning enables computational models consisting of multiple processing layers to learn data representations with various levels of abstraction [26]. This technology has significantly advanced numerous fields, including autonomous driving, medical systems, and agricultural analysis. In this context, deep learning models have emerged for segmenting crop diseases and estimating disease severity levels based on segmentation results. Several noteworthy studies include the following: Ref. [27] introduced a pixel-level segmentation model using an attention mechanism-optimized DeepLabv3+ for accurate grape disease severity estimation. However, their study was limited to simple background disease images, potentially lacking applicability to more complex cases. Ref. [28] developed an improved Mask R-CNN network for the pixel-level segmentation of potato leaves and subsequent grading of late blight. Despite the model’s higher computational load, its performance in severity estimation still requires improvement. Refs. [29,30] presented a two-stage disease segmentation model combining DeepLabv3+ and U-net to sequentially segment diseased leaves and lesions. By calculating the pixel count from the segmentation results, they estimated the disease severity levels. This two-stage approach first extracts diseased leaf instances from images using a leaf segmentation model, followed by lesion segmentation. This method has shown excellent performance in crops like cucumber and corn, but its potential for crops with blurred disease presentations remains to be evaluated.

While these studies have advanced the field significantly, current methods face challenges in assessing cotton verticillium wilt severity: complex backgrounds and limited sample sizes complicate diseased-leaf segmentation; variations in angle and distance affect the location and size of the disease; and cotton verticillium wilt causes shape variations and color changes in leaves, leading to blurred transitions between diseased and healthy areas.

To address these issues, our study introduces a high-precision method for estimating cotton verticillium wilt disease severity. This method aims to provide timely and accurate estimations, facilitating and accelerating disease-resistant cotton breeding efforts. Our study offers the following contributions:

(1) We constructed an image dataset of cotton diseases with complex backgrounds for leaf and lesion segmentation and disease severity level estimation.
(2) We introduced the MobileSAM universal segmentation model, which pre-segments leaf images to enhance performance, especially with limited dataset availability.
(3) We proposed an improved method based on YOLOv8-Seg for segmenting diseased leaves and lesions, addressing challenges such as blurred lesion boundaries and variations in angle and distance. Additionally, we optimized the model parameters and computational complexity.
(4) Through experiments, we validated the effectiveness of the proposed disease severity estimation method. We also developed and deployed a cotton disease severity assessment app for smartphones, which was used in field validation experiments, demonstrating robustness in estimating cotton verticillium wilt severity in field environments.

This paper is organized as follows: Section 2 covers image acquisition, dataset production, the architectures of the methods used, and enhancements to the segmentation model. Section 3 presents the experimental results. Section 4 presents a discussion. Finally, Section 5 concludes this paper.

## 2. Materials and Methods

### 2.1. Materials

#### Image Data Acquisition and Dataset Production

The image dataset for this study was collected from two locations: the cotton fields at the Langfang Research Base of the Chinese Academy of Agricultural Sciences in Hebei Province, China (39°27′55.59″ N, 116°45′28.54″ E), and the Potianyang Base in Yazhou District, Sanya City, Hainan Province, China (18°23′49.71″ N, 109°10′39.84″ E). Data collection occurred between September 2020 and February 2023, under a variety of weather conditions, including clear and overcast skies, at different times of the day—morning, noon, and evening. Images were captured with a Canon EOS 850D digital camera and a Huawei P40 Pro smartphone from distances of 20–50 cm. The images, with a resolution of 4608 × 3456 pixels, were saved in JPG format and included backgrounds such as weeds, soil, leaves, stems, shadows, and human hands. To ensure the accuracy of the dataset, two expert cotton pathologists rigorously diagnosed and confirmed the symptoms in the images. The diagnosis was based on well-established morphological characteristics of Verticillium wilt, including foliar symptoms like leaf chlorosis, necrosis, and wilting. The pathologists carefully differentiated these symptoms from those caused by water stress and other abiotic factors. Water stress symptoms were identified and excluded by observing patterns such as uniform wilting across the entire plant rather than in isolated areas, along with environmental context, such as recent irrigation records and soil conditions. In cases where symptoms were ambiguous, the images were cross-checked with field notes and additional diagnostic methods, such as assessing stem cross-sections for vascular discoloration, which is characteristic of Verticillium wilt.

**Figure 1:**
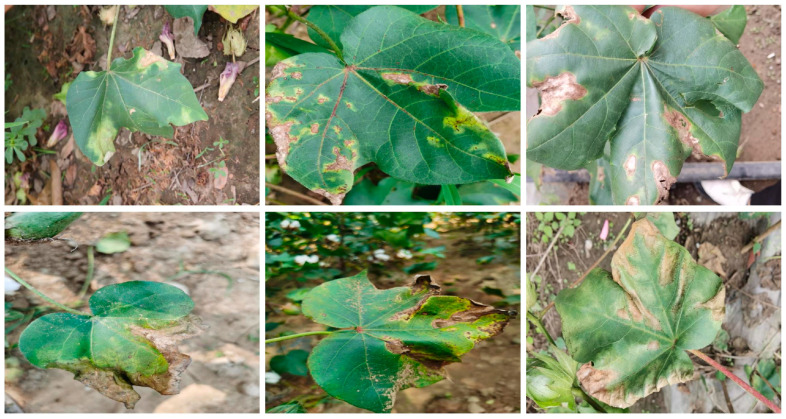
Representative sample images from the cotton Verticillium wilt dataset.

Following image collection, the dataset underwent data augmentation using the Albumentations library (http://github.com/albumentations-team/albumentations (accessed on 22 July 2024)) and the built-in augmentations in YOLOv8. The techniques included horizontal and vertical flips, random rotations, translations, scaling, random cropping, Gaussian noise, brightness and contrast adjustments, and elastic transformations. These augmentations simulated various field conditions, enhancing the model’s robustness and segmentation accuracy under different scenarios. Advanced methods such as grid distortion and optical distortion were also applied to further diversify the training data. For segmentation, we annotated the dataset using LabelMe, creating JSON files with the image size, label names, and points outlining lesions and diseased leaves. This process resulted in 11,310 images of cotton Verticillium Wilt. The dataset was divided into three subsets: 9050 images for training, and 1130 images each for validation and testing. Representative sample images from the dataset are shown in Figure 1.

### 2.2. Methods

#### 2.2.1. Overall Model

One of the primary challenges in automated cotton verticillium wilt severity estimation is accurately segmenting lesions under natural field conditions. Several difficulties arise in this context: (1) Complex backgrounds, such as soil, plastic film, and water pipes, pose significant challenges in segmenting diseased leaves and lesions from images with limited samples. (2) Verticillium wilt in cotton often exhibits excessively blurred lesion boundaries, and variations in angle and distance affect the location and size of the wilt, hindering accurate disease segmentation. (3) Segmentation models require optimization in parameter size and computational complexity to meet deployment needs.

**Figure 2:**
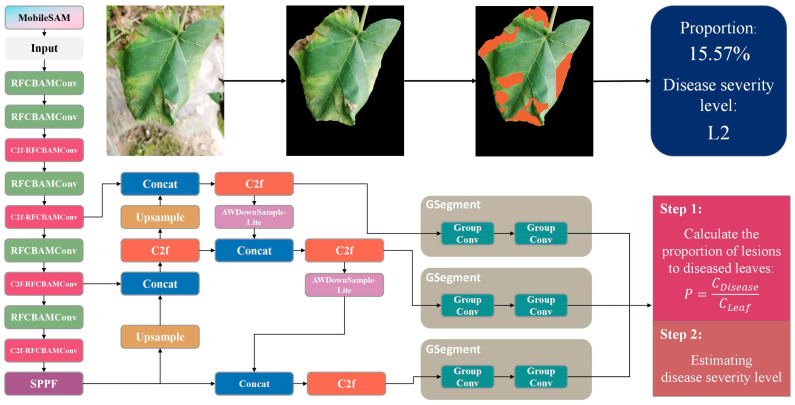
Overall structural diagram of the disease severity estimation model.

In this context, we propose a composite framework, CVW-Etr, for estimating cotton verticillium wilt severity levels under natural field conditions. CVW-Etr is based on the YOLOv8-Seg model and incorporates the RFCBAMConv, C2f-RFCBAMConv, AWDownSample-Lite, and GSegment modules. Additionally, it incorporates YOLOv8-Seg with MobileSAM. The architecture of CVW-Etr is visualized in Figure 2. The framework comprises five main components: input, pre-segmentation, backbone, neck, head, and disease severity level estimation. The methods and enhancements integrated into CVW-Etr are as follows:

(1) Utilizing the MobileSAM suitable for resource-constrained devices, we conducted pre-segmentation on the images, roughly segmenting all leaves in the image and setting the background to black. This enhanced the performance and efficiency of segmentation models trained under a limited sample in a complex field background.
(2) Improved YOLOv8-Seg model for accurate and rapid segmentation: To enhance the segmentation accuracy and speed for cotton verticillium wilt leaves and lesions, several improvements were made to the YOLOv8-Seg model. The RFCBAMConv and C2f-RFCBAMConv modules replaced the Conv and C2f modules in the backbone network, addressing the blurry transitions between healthy and diseased regions. The AWDownSample-Lite module replaced the Conv module in the neck network, handling variations in angle and distance by aggregating information within each receptive field. The GSegment segmentation head, replacing the original segmentation head in YOLOv8-Seg, reduced the model parameters and computational complexity while improving the model’s ability to perceive diseased leaves and lesions at different scales.
(3) The severity levels of cotton verticillium wilt were categorized into six levels, from L0 to L5, based on the proportion of diseased area to lesion area.

#### 2.2.2. MobileSAM

Recently, Meta AI introduced the Segment Anything Model (SAM), a groundbreaking model for image segmentation. SAM is trained on the extensive SA-1B dataset, which includes over 110 million images and 1 billion masks, enabling it to generalize effectively. It utilizes a Transformer-based architecture and is designed to output a segmentation mask for a given input image based on one or several input prompts, such as points, bounding boxes, or segmentation masks [31]. Notably, SAM demonstrates exceptional performance and generalization in common scenes, surpassing the accuracy of prior supervised methods in certain application areas with few samples. Its impact extends across various computer vision applications, including remote sensing segmentation and medical image analysis [32]. In the application scenario of cotton leaf segmentation, the complex backgrounds in cotton fields, including soil, plastic film, and water pipes, pose challenges for visual feature extraction in cotton leaf segmentation. The limited sample size has made it challenging to directly segment diseased leaves from images. However, due to SAM’s outstanding performance in zero-shot learning, its application in estimating cotton verticillium wilt disease severity level holds promise for enhancing performance and efficiency.

Unfortunately, SAM tends to segment the entire object or its main parts, which results in unsatisfactory performance when segmenting verticillium wilt on cotton due to the blurry boundary transitions characteristic of this disease. Consequently, SAM can only segment the leaves from images, and struggles to separate the lesions from the diseased leaves. Furthermore, according to the technical specifications for identifying verticillium wilt resistance in cotton and the experience of disease-resistant cotton researchers, the area of the leaf stem is typically not considered in verticillium wilt disease severity level estimation. However, SAM fails to distinguish subtle visual cues like leaf stems, significantly impacting the accuracy of verticillium wilt disease severity level estimation in the context of disease-resistant cotton breeding. Combining SAM with specialized segmentation models like YOLO-Seg, which has been fine-tuned and optimized to address this task, appears to be a more appropriate approach.

Additionally, its practical applications remain challenging in segmenting diseased leaves. The primary issue stems from the substantial computational resources required by the Transformer (ViT) models, which are integral to SAM’s architecture. Further optimization is needed to meet the deployment requirements on edge computing devices. To address this challenge, ref. [33] proposed a “decoupled distillation” approach to distill the ViT decoder of SAM, resulting in a lightweight version known as MobileSAM. MobileSAM performs satisfactorily on resource-constrained devices such as edge computing devices. Considering these advantages and disadvantages, to ensure the speed and accuracy of CVW-Etr, it relies on MobileSAM for image pre-segmentation. We conducted pre-segmentation on images, roughly segmenting all leaves in the image and setting the background to black, which achieved one-step segmentation of diseased leaves and lesions.

#### 2.2.3. YOLOv8-Seg

YOLOv8s-Seg, developed by Ultralytics, represents the latest advancement in the YOLO (You Only Look Once) series tailored for segmentation tasks, debuting on 10 January 2023 [34]. Drawing inspiration from the principles of the YOLACT network, it comprises three core components—backbone, neck, and head. The model employs a CSPDarknet feature extractor for its backbone and augments it with a C2f module instead of the traditional YOLO neck architecture. Following the C2f module, two segmentation heads predict segmentation masks for the input image. Remarkably, the YOLOv8-Seg model has set new benchmarks in segmentation performance while maintaining exceptional speed and efficiency [35]. Therefore, this paper adopts YOLOv8-Seg as the baseline and proposes further enhancements based on this model.

#### 2.2.4. RFCBAMConv Module and C2f-RFCBAMConv

In images captured from different angles and distances, the location and size of cotton verticillium wilt vary. If convolutional operations use the same parameters in each receptive field to extract information without considering differential information from different locations and the importance of each feature, it can limit the model’s performance in segmenting diseased leaves and lesions, thus affecting the speed and accuracy of disease severity level estimation. While attention mechanisms can address the parameter-sharing issue in convolutional operations, they fail to emphasize the importance of each feature within the receptive field, resulting in insufficient information in the generated attention maps for large-scale convolutions. Additionally, spatial attention mechanisms such as CBAM (the Convolutional Block Attention Module) and CA (Channel Attention) introduce excessive convolution operations and computational burden, rendering them unsuitable for cotton verticillium wilt-diseased leaf and lesion segmentation.

**Figure 3:**
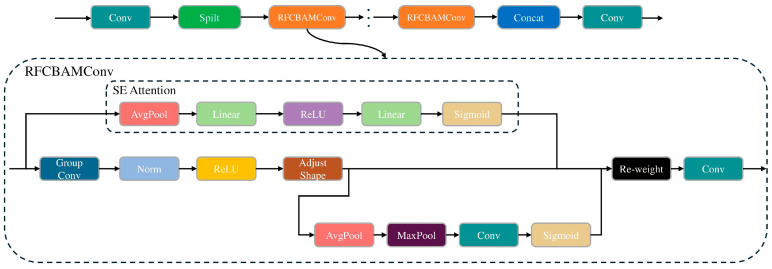
Architectural Diagram of C2f-RFCBAMConv and RFCBAMConv modules.

To address these challenges, this study proposes the RFCBAMConv module, which integrates a spatial attention CBAM focusing on receptive field features with convolution operations. This module not only emphasizes the importance of various features within the receptive field, but also resolves the parameter-sharing issue of convolutional kernels, with only a small increase in the parameters and computational cost. The specific structure of the RFCBAMConv module is illustrated in Figure 3. Regarding attention to receptive field features, the RFCBAMConv module assigns different weights to each position and feature channel within the receptive field using a receptive field weight matrix, thereby adjusting the weight distribution of different features within different receptive fields to highlight important disease details. Concerning parameter sharing, RFCBAMConv adaptively adjusts the shape and scope of the receptive field based on the size of the convolutional kernel, enabling more flexible adjustment of convolutional kernel parameters and providing different processing methods for different regions. Larger receptive fields are utilized to capture global information for heavily affected or larger areas of damage, while smaller receptive fields are generated for lightly affected or smaller targets to enhance the segmentation accuracy of small target lesions. Meanwhile, to further emphasize the detailed features of cotton verticillium wilt and reduce information loss, we integrated the RFCBAMConv module with the C2f module, proposing the C2f_RFCBAMConv module, which replaces the bottleneck in the C2f module with RFCBAMConv. The specific structure is shown in Figure 3.

We integrated the RFCBAMConv and C2f_RFCBAMConv modules into the backbone network of YOLOv8-Seg, replacing the original Conv and C2f modules. This enhancement significantly improves the model’s segmentation performance, particularly for cotton Verticillium wilt lesions. RFCBAMConv adaptively adjusts the receptive field to capture multi-scale lesion features, optimizing feature weighting for both small and large diseased areas. Paired with C2f_RFCBAMConv, the model retains and refines crucial features, enhancing segmentation accuracy in complex field conditions.

#### 2.2.5. AWDownSample-Lite

Cotton verticillium wilt disease induces shape variations and color changes in leaves, often leading to blurred transitions between diseased and healthy areas. Moreover, natural leaf changes, aging, or other physiological factors may resemble disease-induced alterations, making it challenging for models to accurately capture disease shapes and boundaries. The neck section of the YOLOv8-Seg model is crucial for integrating features from different scales. However, its downsampling module filters out what it deems unimportant information during downsampling, affecting the effective extraction of leaf and lesion features of cotton verticillium wilt disease.

**Figure 4:**
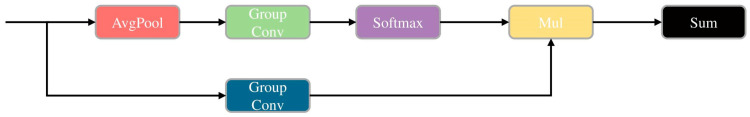
Architecture of AWDownSample-Lite Module.

To address the issue of information loss concerning diseased leaves and lesion features and achieve high-precision models, this study proposes the AWDownSample-Lite module. Inspired by the principle of enlarging the receptive field in the RFAConv design, this module adjusts the receptive field while maximizing the extraction of diseased leaf and lesion features. The specific structure is illustrated in Figure 4, and its implementation steps are as follows:

(1) Input the feature.
(2) Extract global information from the input feature through the AvgPool operation.
(3) Extract information within the receptive field through the Group Conv operation.
(4) Emphasize the importance of each feature within the receptive field through the SoftMax operation.
(5) Fuse the extracted features with the spatial features of the receptive field and utilize them for adjusting the convolutional parameter weights.
(6) Output the feature.

The AWDownSample-Lite module aggregates feature information within each receptive field. This choice ensures that both the global and local context are preserved, helping the model differentiate between disease-induced leaf changes and natural variations due to factors like aging or environmental stress. We integrated the AWDownSample-Lite module into the neck network of the YOLOv8-Seg model, replacing the Conv modules. This enhancement significantly improves the effective extraction of cotton verticillium wilt disease features by the segmentation model, thereby enhancing the accuracy of cotton verticillium wilt disease leaf and lesion segmentation.

#### 2.2.6. GSegment

The YOLOv8-Seg model introduces a decoupled head mechanism, separating convolutional layers from fully connected layers. This technique utilizes the output features from the neck network to predict the category and position of targets through different branches. However, it includes twelve 3 × 3 Conv modules, which, while aiding in improving model convergence and accuracy, introduce a significant number of additional parameters and computational costs.

To enhance computational efficiency, we propose the GSegment module. Inspired by the group convolution design principle of AlexNet, the GSegment module divides convolutional kernels and input feature maps into g groups simultaneously. Within each group, the operation of convolving input feature maps with convolutional kernels is referred to as Group Conv. Group Conv divides the input feature maps into g groups, resulting in a significant reduction in parameters and computational complexity. Additionally, the coupling between the feature maps obtained through different convolution paths is low, with varying attention to primary features. This helps enhance the perception of diseased leaves and lesions of different scales and shapes. The GSegment module replaces the 3 × 3 Convolution in YOLOv8-Seg with two 3×3 Group Convolutions. This improvement significantly reduces the parameter count and computational complexity of the segmentation model.

#### 2.2.7. Severity Level Estimation Method

CVW-Etr estimates the severity levels of cotton verticillium wilt disease by calculating the proportion of lesion area to diseased leaf area. Following technical specifications for evaluating the resistance of cotton verticillium wilt disease [36] and expert recommendations from disease-resistant cotton breeding specialists, severity levels are categorized into six levels, ranging from L0 to L5, as illustrated in Table 1. The intervals corresponding to the proportion of lesion area to diseased leaf area define the severity level.

The specific estimation steps are as follows:

(1) Segmenting cotton verticillium wilt-diseased leaves and lesions.
(2) Calculating the number of pixels of the diseased leaves and lesions based on the segment result.
(3) Computing the proportion of the number of pixels of lesions to diseased leaves, which serves as the basis for grading the severity level of cotton verticillium wilt disease. The calculation formula is presented as Equation (1):

(1) $$P=\frac{C_{\mathrm{Disease}\;}}{C_{\mathrm{Leaf}}}$$

$C_{\mathrm{Disease}\;}$ represents the number of pixels in the segmented lesions, $C_{\mathrm{Leaf}}$ represents the number of pixels in the segmented diseased leaves, and P denotes the proportion of lesions to diseased leaves.

(4) Estimating the cotton verticillium wilt disease severity level based on the proportion of lesions to diseased leaves.

#### 2.2.8. APP Development

In large-scale assessments of cotton Verticillium wilt, evaluating numerous cotton varieties requires surveyors to complete assessments quickly and sync the results to the Cloud for further analysis. Processing data solely on smartphones proved inefficient, leading to increased wait times. Additionally, at tropical cotton breeding sites, where temperatures often exceed 25 °C, field tests revealed that running models locally caused smartphone processors to overheat and even shut down, significantly slowing the survey process and reducing efficiency. To address this, we developed an application for estimating cotton disease severity, implementing a solution that transfers data via HTTPS to a server or edge computing device, improving processing speed and enhancing system stability.

**Figure 5:**
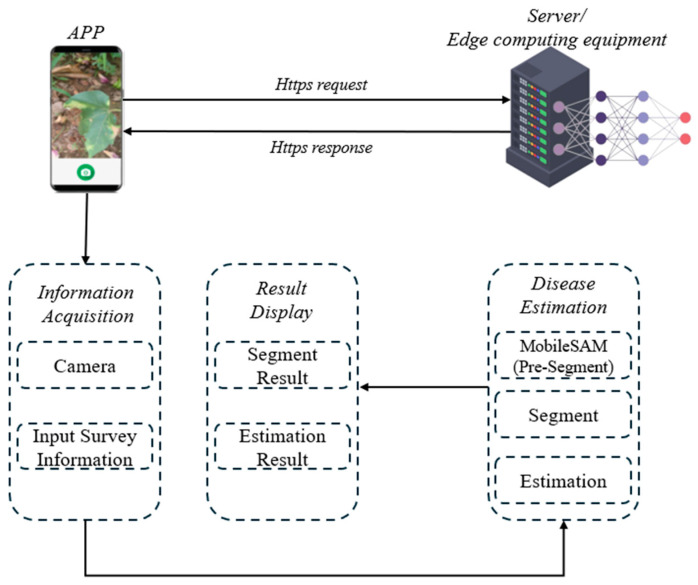
System architecture of the disease severity estimation app.

This application consists of a smartphone terminal and a server terminal, as shown in Figure 5. The smartphone terminal, built on the Uni-app framework, includes two modules: Information Acquisition and Result Display. In the Information Acquisition module, users can capture images of diseased cotton plants and input relevant data, such as the cotton variety, field details, and survey information. These inputs are automatically uploaded to the server via HTTPS for processing. The Result Display module allows users to filter and query the processed results based on criteria such as cotton variety, field location, and disease severity, providing clear and detailed disease assessments.

The server terminal, built on the Micronaut framework, performs key tasks such as segmenting diseased leaves and lesions and estimating disease severity. Once processing is complete, the results are transmitted back to the app for user access.

### 2.3. Model Training Procedures

This experiment was conducted on a Dell desktop workstation running Windows 11, equipped with a 12th Gen Intel Core i5-12500 processor (3.00 GHz), 32 GB RAM, and a 1 TB SSD. GPU acceleration was utilized through an NVIDIA GeForce RTX 3080 (10 GB memory). The software environment included Python 3.7.16, PyTorch 1.7.0, Torchvision 0.8.2, and CUDA 11.0.

The training process spanned 200 epochs, with an early stopping mechanism triggered if performance stagnated for 50 consecutive epochs to prevent overfitting. A batch size of 8 was used, and an Adam optimizer was applied with an initial learning rate of 1 × 10^−3^, which decayed to 1 × 10^−5^. The momentum was set at 0.937 with no weight decay, and the input image resolution was 640 × 640 pixels.

To prevent errors caused by overlapping segments in diseased-leaf segmentation, the overlap_mask parameter was set to False, ensuring overlapping areas were overridden rather than excluded. Additionally, we set the mask_ratio to 1, allowing the segmentation mask to train at the original resolution, enhancing segmentation accuracy for diseased leaves and lesions.

## 3. Results

### 3.1. Performance Evaluation

To evaluate the model’s performance, we used several metrics, including Precision, Recall, mAP@0.5, the number of parameters (Params), and computational costs (FLOPS).

Precision is the ratio of correctly predicted positive samples to all predicted positive samples, and is calculated as follows:

(2) $$Precision=\frac{TP}{TP+FP}$$

where TP denotes true positives and FP denotes false positives.

Recall measures the proportion of actual positive samples correctly identified by the model, calculated as follows:

(3) $$Recall=\frac{TP}{TP+FN}$$

where FN represents false negatives.

mAP (mean average precision) is calculated from a precision–recall curve and defined as follows:

(4) $$mAP=\frac{\sum_{i=1}^{N}\;APi}{N}$$

Here, mAP@0.5 refers to the average AP when the Intersection over Union (IoU) threshold is 0.5.

The number of parameters (Params) reflects the model’s complexity and is given as follows:

(5) $$\mathrm{Param}\;=\sum \left(K\times K\times C_{\mathrm{in}\;}\times C_{\mathrm{out}\;}\right)$$

where K is the convolution kernel size, and $C_{\mathrm{in}\;}$ and $C_{\mathrm{out}\;}$ are the input and output channels, respectively.

FLOPS (Floating-Point Operations Per Second) measures the computational costs, and is calculated as follows:

(6) $$FLOPs=\sum \left(K\times K\times C_{\mathrm{in}\;}\times C_{\mathrm{out}\;}\times H\times W\right)$$

where H × WH × W is the size of the output feature map.

### 3.2. Segmentation Performance of Diseased Leaves and Lesions

Table 2 illustrates the segmentation performance of diseased leaves and lesions using CVW-Etr. The mean average precision (mAP) for diseased-leaf segmentation reaches 99.5%, surpassing the results reported in the existing literature for field environments [37,38,39]. This high performance is attributed to CVW-Etr’s integration of YOLOv8-Seg with MobileSAM, leveraging MobileSAM’s exceptional performance with limited sample sizes, and the fine-tuning and optimization of YOLOv8-Seg. For lesion segmentation, CVW-Etr incorporates the RFCBAMConv, C2f-RFCBAMConv, AWDownSample-Lite, and GSegment modules to handle lesions with blurred transitions effectively, resulting in high-precision segmentation.

### 3.3. Ablation Experiment

To further illustrate the effectiveness of the proposed enhancement method, we conducted ablation experiments using YOLOv8-Seg as the baseline model to estimate the role of each module in detail. The experimental results are summarized in Table 3.

Impact of RFCBAMConv, C2f-RFCBAMConv, and AWDownSample-Lite: Integrating RFCBAMConv, C2f-RFCBAMConv, and AWDownSample-Lite into the YOLOv8-Seg model resulted in a 1.2% increase in mAP. These modules improve model accuracy by handling blurry transitions between healthy and diseased regions and addressing variations in angle and distance during image collection.

Impact of GSegment: After integrating GSegment, the model’s parameter count and computational complexity decreased by 21.21% and 19.84%, respectively, while mAP increased by 0.7%. This suggests that GSegment effectively reduces model parameters and computational complexity, optimizing the model’s performance in accurately estimating cotton verticillium wilt severity.

Overall Effects: The enhanced version of YOLOv8-Seg, incorporating the RFCBAMConv, C2f-RFCBAMConv, AWDownSample-Lite, and GSegment modules, outperformed the original YOLOv8-Seg in terms of mAP, computational complexity, and parameter count. The improved YOLOv8-Seg achieved a 1.9% increase in mAP@0.5, reduced the parameter count by 18.75%, and lowered the computational complexity by 15.83%.

### 3.4. Performance Comparison with the State-of-the-Art Segmentation Models

**Figure 6:**
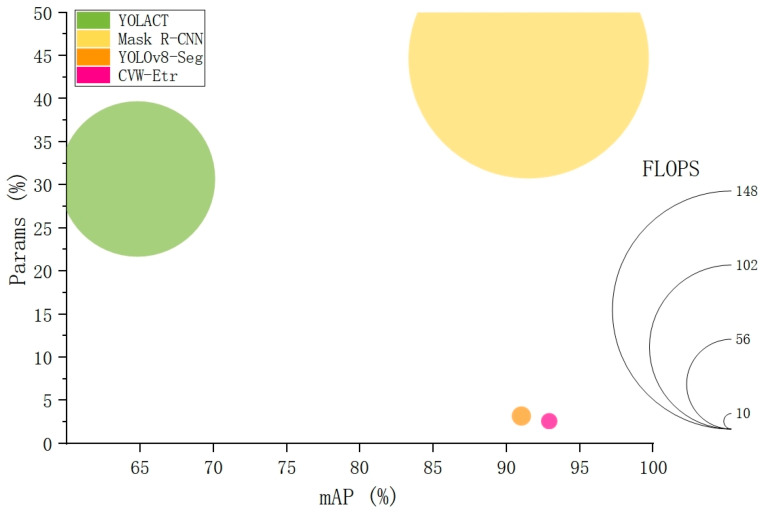
Comparison of segmentation performance across different models.

To assess the effectiveness of our proposed method, we performed comparative experiments against well-known instance segmentation models, including YOLACT, Mask R-CNN, and YOLOv8-Seg. The same dataset was used for all models, comprising 9050 training images, 1130 validation images, and 1130 test images. Consistent experimental conditions were applied across all models to ensure a fair comparison. The results are presented in Figure 6 and Table 4.

CVW-Etr achieves a mAP@0.5 of 92.9%, outperforming YOLACT, Mask R-CNN, and YOLOv8-Seg in terms of segmentation accuracy. Additionally, our proposed model exhibits lower FLOPS and parameters, specifically 10.1G and 2.6M, respectively.

Several factors contribute to these results. Firstly, Mask R-CNN, a classic two-stage instance segmentation model, achieves high segmentation accuracy but requires a higher parameter count and increased computational resources, making it challenging to deploy on resource-constrained devices. Secondly, YOLACT, a one-stage instance segmentation model, excels in terms of parameter count and computational efficiency but lacks accuracy due to its insufficient prototype mask response. Finally, compared to the original YOLOv8-Seg, CVW-Etr achieves a reduction in both model size and computational costs while preserving comparable segmentation accuracy. This improvement is primarily attributed to the integration of the RFCBAMConv, C2f-RFCBAMConv, AWDownSample-Lite, and GSegment modules.

**Figure 7:**
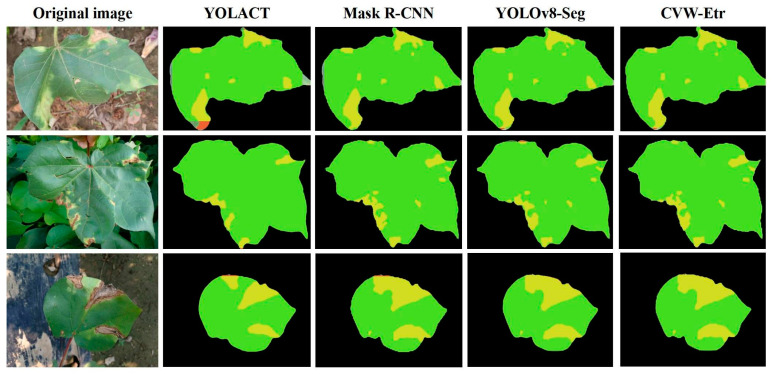
Comparison of segmentation results from different models.

To further substantiate the performance of the CVW-Etr model, we randomly selected segmentation results from various instance segmentation models, as displayed in Figure 7. In these visualizations, green represents the segmentation of diseased leaves, yellow represents the segmentation of lesions, and orange or the absence of a mask indicates missegmentation.

In summary, our results indicate that the proposed model outperforms current mainstream instance segmentation models in terms of three key aspects: model size, detection accuracy, and detection speed. This suggests that CVW-Etr achieves an optimal trade-off between accuracy and speed, making it suitable for estimating the severity levels of cotton verticillium wilt.

### 3.5. Severity Level Estimation Results

**Figure 8:**
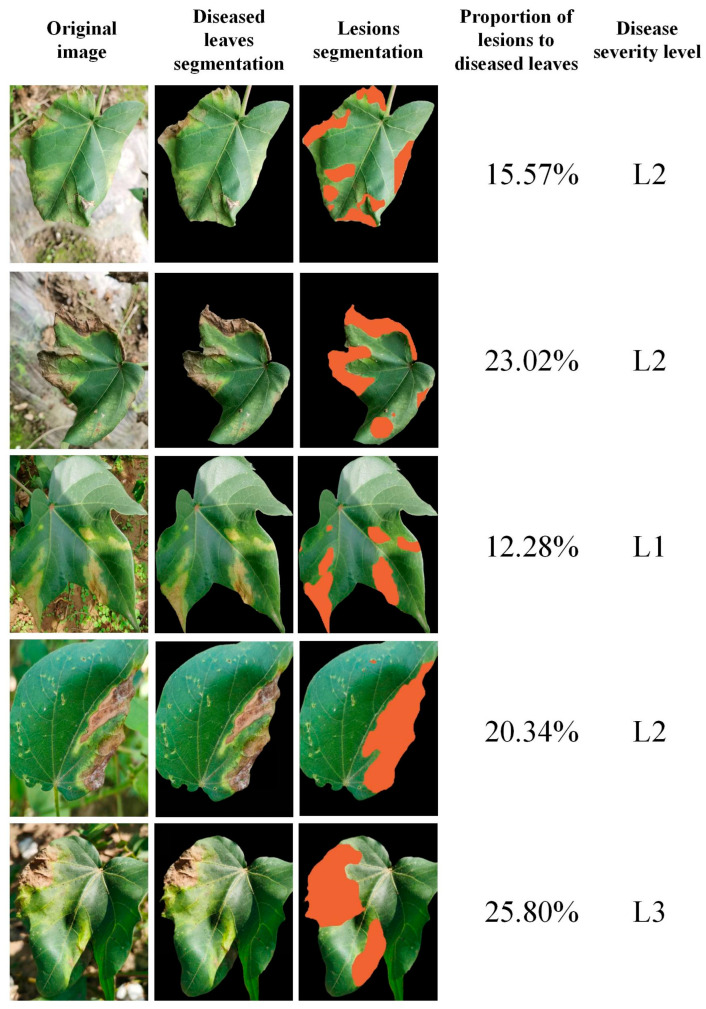
Visual comparison of disease severity estimation results.

This study utilized 113 non-augmented images of cotton verticillium wilt disease to compare the severity levels estimated by the proposed method with those manually estimated by disease-resistant cotton breeding experts, thereby computing the model’s estimation accuracy. The experimental results are presented in Table 5. To further substantiate the performance of the CVW-Etr model, we randomly selected segmentation results from all testing samples, as displayed in Figure 8. In these visualizations, the second column shows the segmentation results for diseased leaves (non-black areas), and the third column highlights the lesion segmentation results with orange masks.

To further validate the model, additional tests were conducted to evaluate its consistency across various environmental conditions and leaf appearances. The CVW-Etr model was tested on images with different lighting conditions, backgrounds, and leaf orientations, demonstrating consistent accuracy and robustness. Consequently, CVW-Etr proves to be reliable in estimating the severity levels of cotton verticillium wilt disease.

### 3.6. Field Validation Experiment

To validate the effectiveness of the proposed method, a field validation experiment was conducted from 10 to 17 December 2023 at the Potianyang Base in Yazhou District, Sanya City, Hainan Province, China. The validation involved 24 participants, including disease-resistant cotton breeding practitioners, experts, local cotton farmers, and graduate students specializing in crop protection or disease-resistance breeding.

Participants were divided into two groups: a manual estimation group consisting of four practitioners and local farmers who relied on their experience and standards to manually estimate cotton verticillium wilt severity, and an automatic estimation group comprising four graduate students who used the developed application for automated estimation. The validation process was as follows:

(1) Both groups independently estimated verticillium wilt severity in 300 cotton plants. In each cotton plant, eight leaves were inspected from top to bottom.
(2) The time taken for estimation was recorded for each group.
(3) Sixteen disease-resistant cotton breeding experts conducted secondary estimations and evaluated the estimation accuracy of each group.

The experimental results, presented in Table 6, indicated that the automatic group required significantly less time than the manual group. Due to the large number of cotton plants assessed, the manual group’s efficiency decreased, and estimation errors increased after 20 min. Conversely, the automatic group demonstrated higher efficiency and accuracy. These results unequivocally show that CVW-Etr is highly suitable for the accurate and large-scale estimation of cotton verticillium wilt severity, particularly in the context of disease-resistant cotton breeding.

## 4. Discussion

### 4.1. Contributions of This Study

Estimating cotton disease severity using deep learning has been extensively researched with two primary methods: spectral analysis and UAV-based approaches. However, both methods have notable limitations.

Previous studies [16,19,40] have primarily employed spectrometry-based methods. While these methods are effective for estimating disease severity, they face challenges in real field conditions, such as varying illumination and soil interference, which can reduce the accuracy and reliability of the spectral data. Furthermore, the high cost of spectrometric equipment and its susceptibility to environmental conditions limit its use in mobile applications.

Similarly, studies by [41,42,43] used UAV-based approaches to estimate cotton disease severity. However, these methods often lack the precision necessary for estimating the severity level of individual breeding materials, which is critical in disease-resistance breeding applications. Despite extensive research using these methods, there remains a gap in addressing the segmentation of diseased leaves and lesions for precise cotton disease severity estimation, especially in the context of disease-resistance breeding. This study aims to fill this gap with the introduction of CVW-Etr, which offers the following advantages:

(1) Enhanced Performance and Efficiency: To improve segmentation performance with limited datasets in complex field conditions, CVW-Etr utilizes MobileSAM for image pre-segmentation. This helps to enhance model performance, especially when only a small dataset is available.
(2) Integrated Segmentation Models: To address challenges like blurry transitions between healthy and diseased regions, variations in angles and distances, and the need for model optimization, CVW-Etr incorporates modules such as RFCBAMConv, C2f-RFCBAMConv, AWDownSample-Lite, and GSegment to optimize the YOLOv8-Seg model for more accurate segmentation.
(3) High-Precision Severity Estimation: Based on the segmentation results of diseased leaves and lesions, CVW-Etr classifies cotton Verticillium wilt severity into six levels (L0 to L5) by calculating the ratio of diseased leaf area to lesion area. This method meets the high precision requirements for severity estimation in disease-resistance breeding applications.

### 4.2. Limitations and Future Prospects

While the proposed method has shown promising results, several limitations must be addressed in future research:

(1) Challenges with Severe Infections: Cotton leaves severely infected with Verticillium wilt (L4 or L5) often exhibit significant damage or curling. Although CVW-Etr can provide accurate estimates due to large lesion areas, the segmentation of severely damaged leaves may introduce errors. Future work will focus on improving severity estimates for these cases by incorporating image recognition techniques.
(2) Data Limitations: This study used images from Hebei and Hainan provinces for training and validation, with field verification limited to Hainan. Due to the limitation in publicly available datasets for cotton Verticillium wilt, in future research, we will expand our datasets by integrating models [44] with agricultural inspection robots to collect data under diverse conditions. Additionally, we plan to involve human experts to verify and correct model predictions and incorporate techniques such as conditional Generative Adversarial Networks (GANs) to generate synthetic images, which will augment our dataset and improve model robustness.
(3) Scope of CVW-Etr: Currently, CVW-Etr only estimates the severity of cotton Verticillium wilt and does not address other cotton diseases. Future research will focus on expanding the model to cover additional cotton diseases.

Despite these limitations, CVW-Etr provides a valuable technical reference for estimating cotton Verticillium wilt severity in complex field environments.

## 5. Conclusions

This study presents CVW-Etr, a precise method for estimating cotton verticillium wilt severity. CVW-Etr categorizes severity into six levels (L0 to L5) based on the proportion of segmented lesions to diseased leaves. By integrating MobileSAM with the YOLOv8-Seg model, CVW-Etr enhances performance and efficiency, even with limited samples in complex field backgrounds. It incorporates the RFCBAMConv, C2f-RFCBAMConv, AWDownSample-Lite, and GSegment modules to handle blurry transitions between healthy and diseased regions and variations in angle and distance, and to optimize the model’s parameter size and computational complexity. Our experimental findings demonstrate CVW-Etr’s effectiveness in segmenting diseased leaves and lesions, achieving a mean average precision (mAP) of 92.90% and an average accuracy of 92.92% in disease severity estimation with only 2.6M parameters and 10.1G FLOPS. The main advantages of CVW-Etr over existing methods include its ability to handle complex field backgrounds, its efficiency with limited data, and its lower computational cost. Despite CVW-Etr’s robustness in estimating cotton Verticillium wilt severity and its valuable contributions to disease-resistant cotton breeding, the model currently has some limitations. For instance, it is specifically designed for cotton Verticillium wilt and has not yet been adapted to other cotton diseases or crop types. Future research will focus on expanding these severity estimation methods to cover additional cotton diseases and potentially different crops to broaden the model’s applicability. Additionally, we aim to develop a comprehensive management system for disease-resistant cotton breeding, integrating disease detection, identification, and severity estimation into a unified platform. This system will provide an efficient tool for the accurate management of disease-resistant breeding materials.

## Figures and Tables

**Table 1:** Criteria for estimating disease severity levels in cotton verticillium wilt.

Disease Severity of Leaves	Proportion of Lesions to Diseased Leaves
L0	0
L1	0 < *p* ≤ 0.15
L2	0.15 < *p* ≤ 0.25
L3	0.25 < *p* ≤ 0.40
L4	0.40 < *p* ≤ 0.60
L5	0.60 < *p* ≤ 1.00

**Table 2:** Segmentation performance for diseased leaves and lesions.

Class	Precision	Recall	mAP@0.5	mAP@0.5:0.95
Diseased leaves	97.7%	100%	99.5%	95.6%
Lesions	88.3%	82.0%	86.3%	54.8%
All	93.0%	91.0%	92.9%	75.2%

**Table 3:** Performance comparison of ablation experiment results.

Baseline	RFCBAMConv	AWDownSample-Lite	GSegment	mAP@0.5	FLOPS/G	Params/M
✓				91.0%	12.1	3.2
✓	✓			91.8%	12.7	3.3
✓		✓		91.9%	12.1	4.0
✓			✓	90.7%	9.7	2.6
✓	✓	✓		92.2%	12.7	3.2
✓	✓		✓	91.1%	10.2	2.7
✓		✓	✓	90.8%	9.8	2.6
✓	✓	✓	✓	92.9%	10.3	2.6

**Table 4:** Segmentation performance comparison of different models.

Models	mAP@0.5	FLOPS/G	Params/M
YOLACT	64.8%	96.4	30.7
Mask R-CNN	91.5%	149.0	44.7
YOLOv8-Seg	91.0%	12.0	3.2
CVW-Etr	92.9%	10.1	2.6

**Table 5:** Accuracy of disease severity estimation at different levels.

Disease Severity Level	Number	Correct Estimation	Accuracy/%
L0	20	18	90.00%
L1	20	18	90.00%
L2	20	19	95.00%
L3	14	13	92.86%
L4	19	17	89.47%
L5	20	20	100%
All	113	105	92.92%

**Table 6:** Field validation comparison of manual and CVW-Etr methods.

Method	Accuracy/%	Time/s
Manual	91.33%	1770
Automatic (CVW-Etr)	92.66%	1330

## Data Availability

The model weights, the codes, and any derivatives of the YOLOv8 component in this study are licensed under the Affero General Public License (AGPLv3). In adherence to the AGPLv3 license, the codes, the optimal model, and some of the data that were used and analyzed in this study can be accessed on the following website: https://github.com/cn-panpan/CVW-Etr (accessed on 22 July 2024). The data used to support this study are available from the corresponding author upon request.
